# Lung Metabolism and Inflammation during Mechanical Ventilation; An Imaging Approach

**DOI:** 10.1038/s41598-018-21901-0

**Published:** 2018-02-23

**Authors:** Mehrdad Pourfathi, Maurizio Cereda, Shampa Chatterjee, Yi Xin, Stephen Kadlecek, Ian Duncan, Hooman Hamedani, Sarmad Siddiqui, Harrilla Profka, Jason Ehrich, Kai Ruppert, Rahim R. Rizi

**Affiliations:** 1 Department of Radiology, University of Pennsylvania, Philadelphia, PA USA; 2 Department Electrical and Systems Engineering, University of Pennsylvania, Philadelphia, PA USA; 3 Department of Anesthesiology and Critical Care, University of Pennsylvania, Philadelphia, PA USA; 4 Department of Physiology, University of Pennsylvania, Philadelphia, PA USA; 5 Department of Bioengineering, University of Pennsylvania, Philadelphia, PA USA

## Abstract

Acute respiratory distress syndrome (ARDS) is a major cause of mortality in critically ill patients. Patients are currently managed by protective ventilation and alveolar recruitment using positive-end expiratory pressure (PEEP). However, the PEEP’s effect on both pulmonary metabolism and regional inflammation is poorly understood. Here, we demonstrate the effect of PEEP on pulmonary anaerobic metabolism in mechanically ventilated injured rats, using hyperpolarized carbon-13 imaging. Pulmonary lactate-to-pyruvate ratio was measured in 21 rats; 14 rats received intratracheal instillation of hydrochloric-acid, while 7 rats received sham saline. 1 hour after acid/saline instillation, PEEP was lowered to 0 cmH_2_O in 7 injured rats (ZEEP group) and in all sham rats; PEEP was continued in the remaining 7 injured rats (PEEP group). Pulmonary compliance, oxygen saturation, histological injury scores, ICAM-1 expression and myeloperoxidase expression were measured. Lactate-to-pyruvate ratio progressively increased in the dependent lung during mechanical ventilation at ZEEP (*p* < 0.001), but remained unchanged in PEEP and sham rats. Lactate-to-pyruvate ratio was correlated with hyaline membrane deposition (*r* = 0.612), edema severity (*r* = 0.663), ICAM-1 (*r* = 0.782) and myeloperoxidase expressions (*r* = 0.817). Anaerobic pulmonary metabolism increases during lung injury progression and is contained by PEEP. Pulmonary lactate-to-pyruvate ratio may indicate *in-vivo* neutrophil activity due to atelectasis.

## Introduction

Acute respiratory distress syndrome (ARDS) is a fatal condition that occurs in approximately 10% of patients admitted to the intensive care unit (ICU)^1^, and is characterized by increased alveolar membrane permeability leading to edema, severely impaired gas exchange, hypoxemia and pulmonary neutrophilic infiltration as an innate inflammatory response^2,3^. To minimize the progression of lung injury and inflammation and reduce secondary lung injury due to inspiratory stretch, ARDS patients are managed in the ICU through protective ventilation with low tidal volumes (V_T_, Table 1) and positive-end expiratory pressure (PEEP)^2,4^. Although this strategy has been shown to decrease mortality in more severe ARDS patients^5–7^, regional tissue stress due to poorly recruited atelectasis also has the potential to worsen injury during ventilation^4,8^. What is more, the effects of atelectasis and alveolar recruitment on lung cellularity^9^, regional inflammation^10^ and lung metabolism^11^ are incompletely characterized.

**Table 1:** List of relevant terms and abbreviations.

Parameter	Definition
ARDS	Acute respiratory distress syndrome
V_T_	Tidal Volume
PIP	Peak inspiratory pressure
PEEP	Positive-end expiratory pressure
ZEEP	Zero PEEP
C_dyn_	Dynamic compliance, a measure of lung’s elasticity defined as (V_T_/(PIP-PEEP))
FiO_2_	Fraction of inspired oxygen
Atelectasis	Collapse of the alveoli in the lungs
^18^F-FDG	^18^F-Fluorodeoxyglocuse
PET	Positron emission tomography
HP-MRI	Hyperpolarized magnetic resonance imaging
DNP	Dynamic nuclear polarization, a process to enhance the MR signal by ~10,000-fold
Neutrophils	Most abundant type of white blood cells that are recruited upon injury
MPO	Myeloperoxidase, an enzyme expressed in activated neutrophils
ICAM-1	Intercellular adhesion molecule-1, a protein expressed in endothelial cells and facilitates neutrophilic recruitment
Dependent Lung	The lowest part of the lungs relative to the gravity

Neutrophil recruitment and activation are pivotal in the trajectory of ARDS^12,13^. Positron emission tomography (PET) has shown increased ^18^F-fluorodeoxyglucose (^18^F-FDG) uptake in regions with inflammatory lesions in ARDS patients^11^, indicating increased glycolysis in activated neutrophils^14^. Yet FDG-PET is unable to reveal the ultimate metabolic fate of glucose in the injured lung. Increased trans-pulmonary blood lactate as a result of anaerobic metabolism is correlated with lung injury severity, and is generally attributed to glucose uptake and utilization by activated inflammatory infiltrates^15,16^. Additionally, recent studies suggest that locally elevated lactic acid concentrations might have a role in promoting biological processes in lung tissue such as fibrosis^17^. Therefore, tissue lactate mapping could be used as both a topographic marker of injury severity and to monitor therapeutic response.

Hyperpolarized (HP) carbon-13 magnetic resonance imaging (MRI) is a novel imaging technique that highlights changes in cellularity and metabolic pathways by quantifying downstream metabolites through their unique carbon-13 chemical shift^18^. The temporarily enhanced carbon-13 NMR signal of hyperpolarized agents (~10,000-fold) made possible by dynamic nuclear polarization (DNP)^19^, provides a general molecular imaging probe capable of rapidly and noninvasively interrogating molecular pathways within minutes in animal models^18^ and, more recently, in human subjects^20^.

In previous studies using HP [1-^13^C] pyruvate and its conversion to lactate to assess lung metabolism^21^, we found elevated lactate-to-pyruvate ratio in animal models of acute lung inflammation^22^ and injury^23^. In this study, we used hyperpolarized carbon-13 MRI for assessing the impact recruiting atelectasis on lung anaerobic metabolism during the early progression of lung injury. We tested the hypothesis that recruitment contains anaerobic metabolism in the lung tissue by limiting injury due to atelectasis. Lung mechanics, oxygenation and histological analysis of lung sections supported this hypothesis, suggesting that the increased conversion of pyruvate to lactate in the absence of PEEP is primarily the result of recruitment and activation of neutrophils in the lungs.

## Results

### Hyperpolarized carbon-13

**Figure 1:**
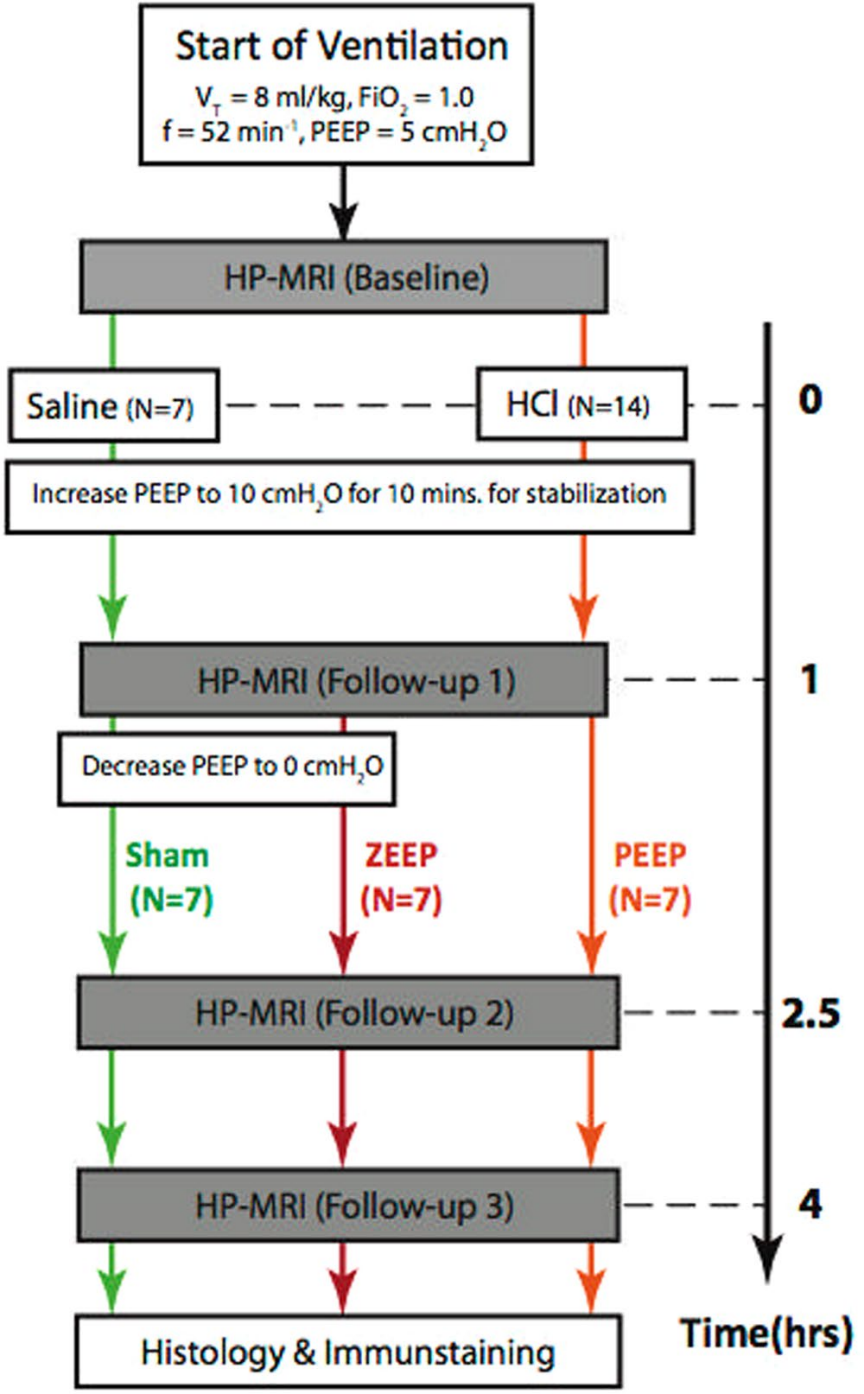
Summary of hyperpolarized carbon-13 imaging studies. A healthy baseline carbon-13 image was initially acquired. Rats received 0.5 ml/kg of either hydrochloric acid (N = 14) or saline (N = 7) 10 minutes after the baseline scan. Immediately after, positive-end expiratory pressure (PEEP) was increased to 10 cmH_2_O during a 10-minute stabilization period and was lowered back to 5 cmH_2_O. After 1 hour, the first follow-up carbon-13 image was acquired. PEEP was then lowered to 0 cmH2O in the rats receiving saline instillation (Sham group) and in seven injured rats (ZEEP group). Subsequent carbon-13 images were acquired 2.5 and 4 hours after acid/saline instillation. (Note that four animals from in the ZEEP group did not receive the third injection. Statistics are corrected for the unbalanced data).

**Figure 2:**
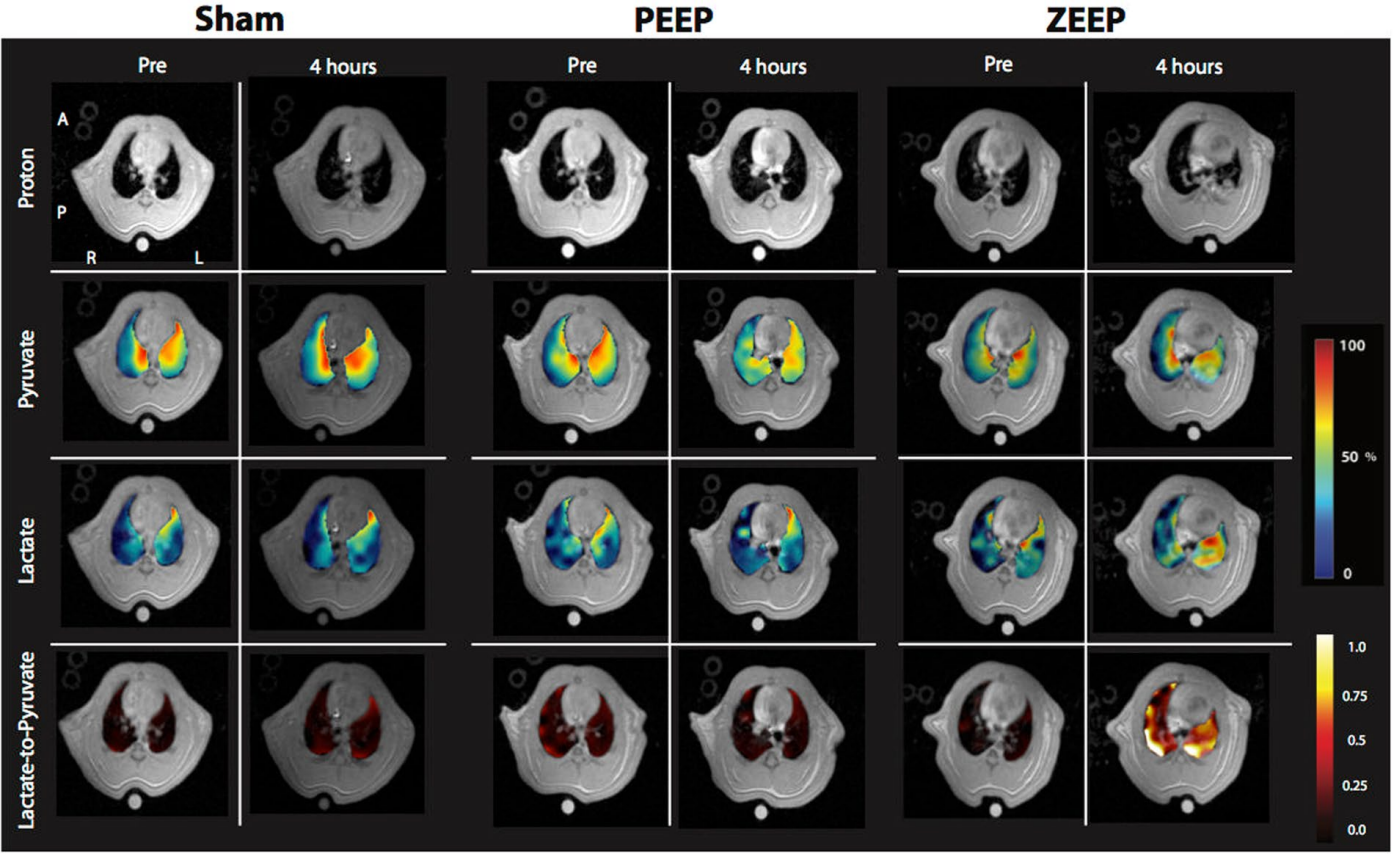
Representative proton images, pyruvate, lactate and lactate-to-pyruvate maps overlaid on their corresponding proton images. The left panels show the images acquired at healthy baseline and 4 hours after acid/saline instillation in a sham rat. The middle and right panels show similar maps for the PEEP and ZEEP rats. Note the dramatic increase in lactate signal and lactate-to-pyruvate ratio in the ZEEP rat 4 hours after acid instillation. The superimposed maps are segmented to show only the values within the lung fields. For display, the pyruvate and lactate maps are normalized to their own maximum intensity for each pulmonary map. Images acquired at the second and third time points are included in the supplementary section.

Figure 1 shows the summary of the imaging experiments. Figure 2 shows representative anatomical and interpolated metabolic images at the first and last time-points for all three groups. The pyruvate signal did not significantly change over time in any of the groups, and was at its most intense in the major vasculature close to the heart. Lactate signal intensity remained stable over time in both sham and PEEP rats, as did the lactate-to-pyruvate maps for these two experimental groups. In contrast, we observed time-dependent increases of lactate signal and lactate-to-pyruvate ratio in posterior lung regions in the ZEEP (zero positive-end expiratory pressure) group, which were co-localized with increased proton signal intensity. Although less pronounced, we also observed a gradual increase in the lactate-to-pyruvate ratio map in the central and anterior lung in ZEEP rats.

**Figure 3:**
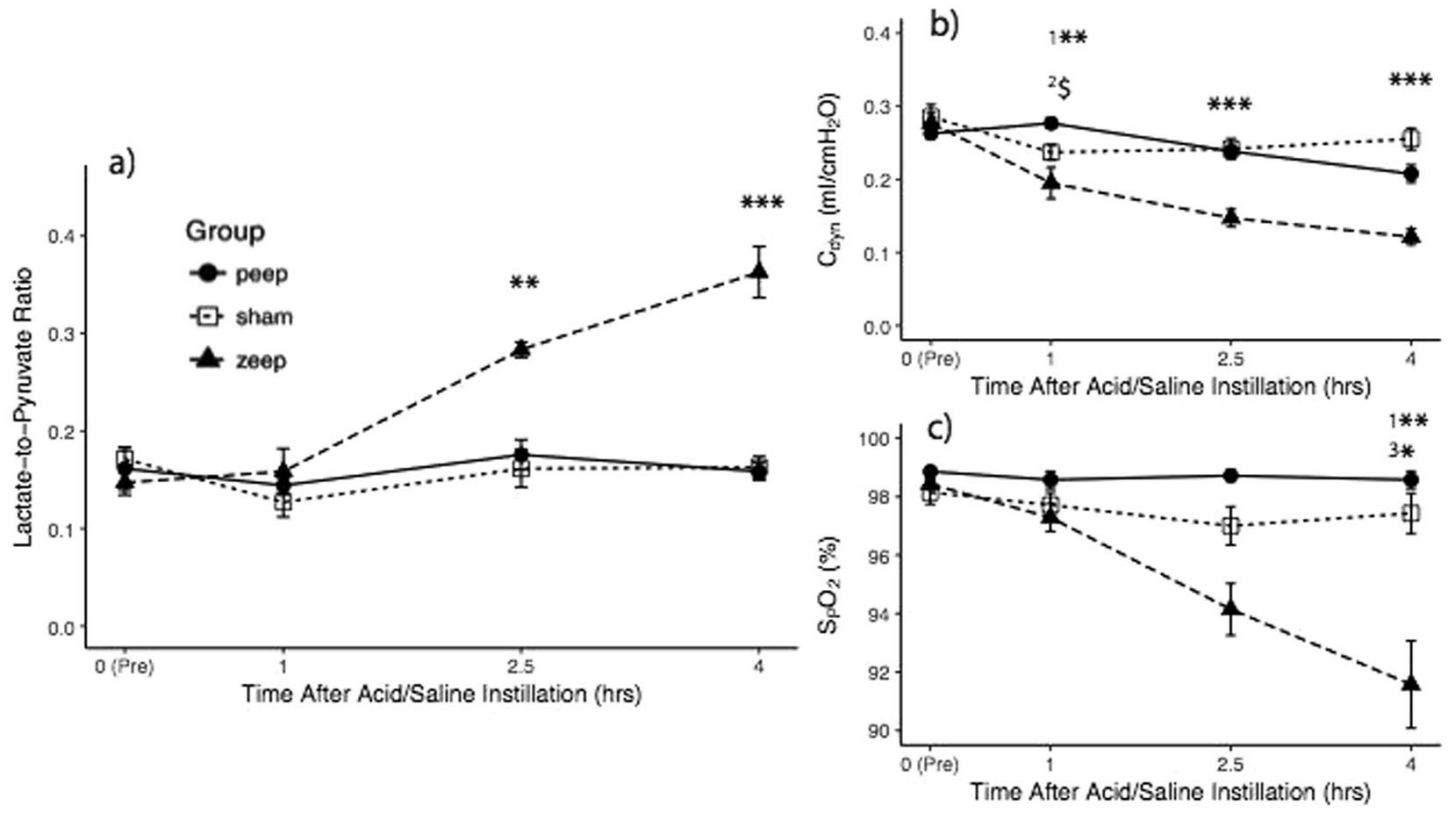
(**a**) Average lactate-to-pyruvate ratio was significantly higher in the ZEEP group than in sham and PEEP groups 2.5 (p < 0.01 for both comparisons) and four hours (p < 0.001 for both comparisons) after acid/saline instillation. There was a no significant difference between the latter two groups at both 2.5 and 4 hours. (**b**) Pulmonary compliance (C_dyn_) declined in the injured rats 1 hour after acid instillation, and continued to decline in the ZEEP group compared to the sham and PEEP groups (p < 0.001). C_dyn_ was not statistically different between sham and PEEP rats during the rest of the experiment. (**c**) Oxygen saturation (S_p_O_2_) was similar among the groups at healthy baseline and 1 hour after acid/saline instillation, but declined over time in the ZEEP group while remaining unchanged in the sham and PEEP groups. Groups were statistically different at 4 hours after acid/saline instillation (χ^2^(2) = 10.004, p = 0.006), with the ZEEP group having significantly lower oxygen saturation than the sham (p = 0.003) and PEEP (p = 0.035) groups. PEEP and Sham groups were not different (p = 0.804). (^$^p < 0.1, *p < 0.05, **p < 0.01, ***p < 0.001), (^1^ZEEP vs. Sham, ^2^PEEP vs Sham, ^3^PEEP vs. ZEEP). (Note that for lactate-to-pyruvate ratio at 2.5 hours, N = 3 for the ZEEP group. For all groups and all time-points N = 7).

Initial ANOVA of the average lactate-to-pyruvate ratio (Fig. 3a) showed discrimination among groups (F_2,74_ = 8.242, *p* < 0.001) and pyruvate injections (F_3,74_ = 7.023, *p* < 0.001). The post-hoc analysis is reported in Table 2: the average lactate-to-pyruvate ratio was similar among groups at both healthy baseline and 1 hour after acid/saline instillation, but was significantly different both 2.5 hours and 4 hours after the instillation of acid/saline. 2.5 hours after acid instillation, lactate-to-pyruvate ratio was significantly higher in the ZEEP group than in sham and PEEP groups, and these differences further increased at 4 hours. There was a no significant difference between the latter two groups at both 2.5 and 4 hours.

**Table 2:** Summary of post-hoc analysis performed using the Tukey honest significant difference test, performed when the results of the one-way ANOVA test (with Bonferroni adjustment for four time points α_adj_. = 0.05/4 = 0.0125) for each time point was significant.

Parameter	Time (hrs)^#^	Group Comparisons								
		PEEP - Sham			ZEEP - Sham			ZEEP - PEEP		
		Δmean	95% C.I.	*p*-value	Δmean	95% C.I.	*p*-value	Δmean	95% C.I.	*p*-value
Lactate-to-Pyruvate Ratio	2.5	−0.014	−0.074, 0.046	0.814	0.107	0.029, 0.186	0.008*	0.122	0.043, 0.200	0.003*
	4	−0.005	−0.088, 0.076	0.921	0.208	0.134, 0.282	<0.001*	0.296	0.131, 0.296	<0.001*
Pulmonary Compliance	1	−0.020	−0.043, 0.003	0.097	−0.036	−0.059, −0.013	0.002*	−0.016	−0.039, 0.007	0.213
	2.5	0.003	−0.038, 0.045	0.944	−0.091	−0.133, −0.049	<0.001*	−0.094	−0.136, −0.052	<0.001*
	4	0.010	−0.029, 0.049	0.596	−0.096	−0.131, −0.061	<0.001*	−0.106	−0.145, −0.067	<0.001*

Initial two-way ANOVA of the average lactate-to-pyruvate ratio showed discrimination among groups (F_2,74_ = 8.242, p < 0.001) and pyruvate injections (F_3,74_ = 7.023, p < 0.001). The average lactate-to-pyruvate ratio was similar among groups both at healthy baseline (F_2,18_ = 0.648, p = 0.535) and 1 hour after acid/saline instillation (F_2,18_ = 0.514, p = 0.513), but was significantly different 2.5 hours (F_2,14_ = 8.843, p = 0.003) and 4 hours (F_2,14_ = 5.533, p < 0.001) after the instillation of acid/saline. The initial ANOVA of the pulmonary compliance (C_dyn_) showed significant inter-group difference (F_2,74_ = 16.56, p < 0.001). C_dyn_ was also significantly different among groups 1 hour (F_2,18_ = 7.866, p = 0.003), 2.5 hours (F_2,18_ = 16.1, p < 0.001) and 4 hours (F_2,18_ = 20.8, p < 0.001) after acid/saline instillation. (^#^Time from the instillation of acid/saline).

### Pulmonary function

The initial ANOVA of compliance (Fig. 3b) and oxygen saturation (Fig. 3c) showed significant inter-group difference (C_dyn_: F_2,74_ = 16.56, *p* < 0.001 and S_p_O_2_: χ^2^(2) = 20.054, *p* < 0.001). Post-hoc analysis is summarized in Table 2: C_dyn_ was also significantly different among groups 1 hour, 2.5 hours and 4 hours after acid/saline instillation. C_dyn_ declined in the injured rats 1 hour after acid instillation, and continued to decline in the ZEEP group compared to sham and PEEP groups. C_dyn_ was not statistically different between sham and PEEP rats during the rest of the experiment.

S_p_O_2_ was similar among the groups at healthy baseline and 1 hour after acid/saline instillation, but declined over time in the ZEEP group while remaining unchanged in sham and PEEP groups. Groups were statistically different at 4 hours after acid/saline instillation (χ^2^(2) = 10.004, *p = *0.006), with the ZEEP group having significantly lower oxygen saturation than the sham (*p* = 0.003) and PEEP (*p* = 0.035) groups. PEEP and sham groups were not different (*p* = 0.804). Heart rate for all rats ranged between 350–400 beats-per-minute.

**Figure 4:**
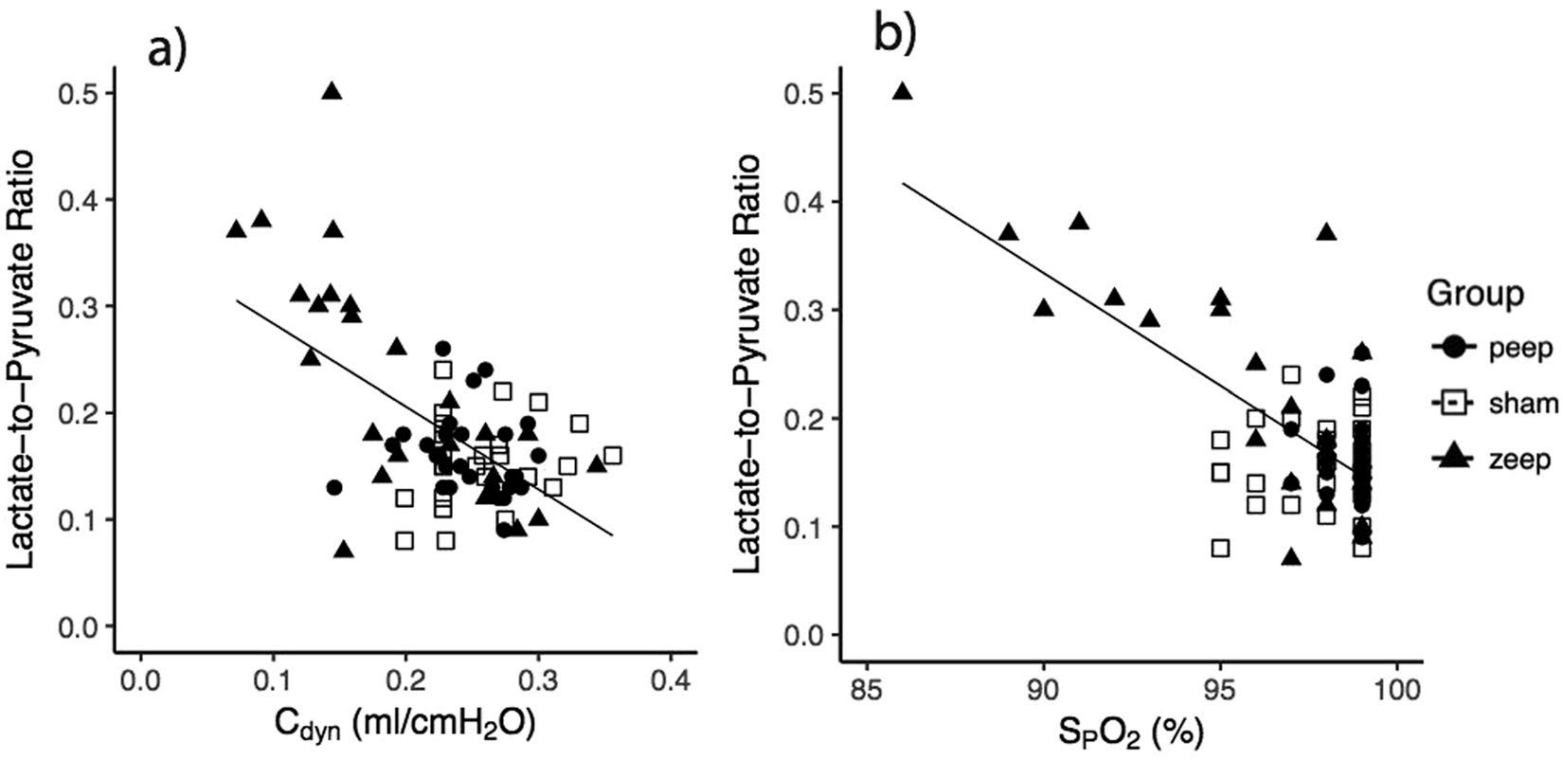
Correlation between average lactate-to-pyruvate ratio and (**a**) pulmonary compliance (r = −0.581, p < 0.001) and (**b**) oxygen saturation (r = −0.687, p < 0.001).

Oxygen saturation and pulmonary compliance were significantly and negatively correlated with average lactate-to-pyruvate ratio (Fig. 4a,b, Table 3).

**Table 3:** Pearson correlation coefficients between the global lactate-to-pyruvate ratio and other experimental parameters.

	Correlation between Lactate-to-Pyruvate Ratio and other Parameters			
	Parameter	***r***	**95% C.I**.	***p*** **-value**
Final imaging data only	Pulmonary Compliance	−0.581	−0.709, −0.413	<0.001*
	Oxygen Saturation	−0.687	−0.789, −0.553	<0.001*
	Infiltration Score	0.509	0.100, 0.771	0.018
	Alveolar Structure Damage	0.485	0.068, 0.758	0.025
	Hyaline Membrane Score	0.612	0.245, 0.825	0.003*
	Edema Score	0.663	0.325, 0.851	0.001*
	ICAM-1 Expression	0.782	0.529, 0.907	<0.001*
	Myeloperoxidase Activity	0.817	0.589, 0.921	<0.001*

Pulmonary compliance and oxygen saturation was measured at the beginning of carbon-13 imaging. Histological features were only correlated with the last imaging time-point. Bonferroni correction was applied for 8 univariate correlation tests (significance for adjusted α_adj._ = 0.05/8 = 0.00625).

### H&E Immunohistochemistry

**Figure 5:**
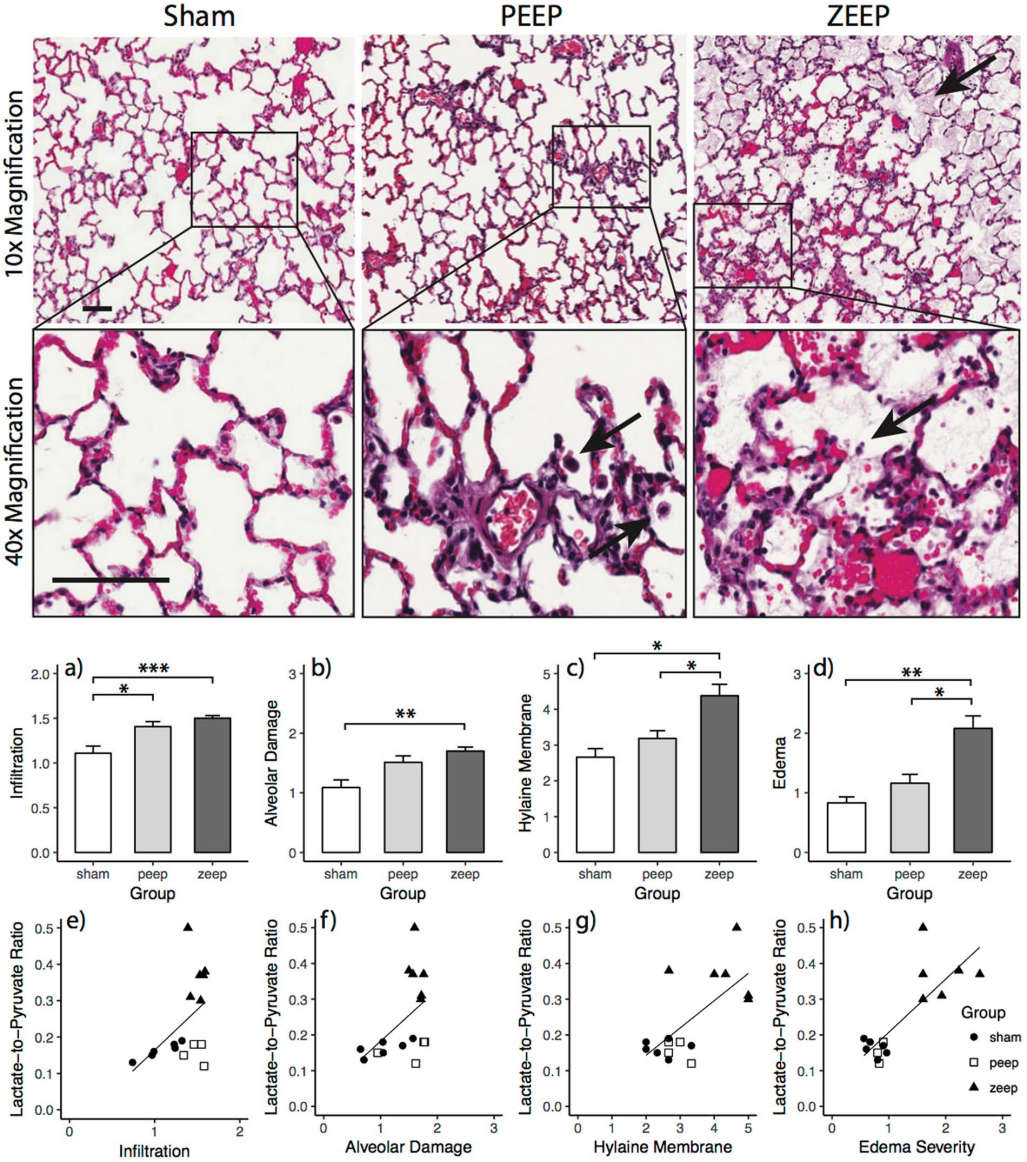
(Top) Hematoxylin and Eosin (H&E) stained lung sections of rats from each group. Representative images shown are at 10× and 40×. In ZEEP lungs, arrows show the presence of fluid within the alveolar space. Solid arrows delineate infiltrates and debris. In lungs subjected to PEEP, alveolar structure is more intact. Infiltrates are shown by solid arrows. The scale bar is 100 µm. (Middle) Average injury scores for various morphological features for different groups. ANOVA showed a significant difference among groups (infiltration: χ^2^(2) = 11.273, *p* = 0.003, alveolar damage: χ^2^(2) = 10.000, *p* = 0.007, hyaline membrane deposition: χ^2^(2) = 10.678, *p* = 0.005 and edema: χ^2^(2) = 12.154, *p* = 0.002). Infiltration (**a**) and alveolar damage scores (**b**) were significantly higher in the ZEEP group than in the sham group (*p* = 0.001, *p* = 0.012), while infiltration was significantly higher in the ZEEP group than the PEEP group (*p* = 0.035). Hyaline membrane deposition (**c**) and edema severity (**e**) were both significantly higher in the ZEEP group relative to the PEEP (*p* = 0.048, *p* = 0.041) and sham (*p* = 0.014, *p* = 0.006) groups. (Bottom) Among the histological features, hyaline membrane deposition (*r* = 0.612, *p* = 0.003) (**g**) and edema severity (*r* = 0.663, *p* = 0.001) (**h**) were strongly correlated with lactate-to-pyruvate ratio. (*p < 0.05, **p < 0.01, ***p < 0.001).

ZEEP lungs displayed evidence of lung injury (Fig. 5), including structural damage, with edema and hyaline membrane on the alveolar wall; aggregates were found in the alveoli, likely representing sloughed pneumocytes. Both ZEEP and PEEP lungs had alveolar infiltrates. There was no evidence of injury in the sham lungs; alveoli were lined with flattened healthy epithelial cells, with minimal inflammatory cells present.

ANOVA showed a significant difference among groups (infiltration: χ^2^(2) = 11.273, *p* = 0.003, alveolar damage: χ^2^(2) = 10.000, *p* = 0.007, hyaline membrane deposition: χ^2^(2) = 10.678, *p* = 0.005 and edema: χ^2^(2) = 12.154, *p* = 0.002). Infiltration (Fig. 5a) and alveolar damage scores (Fig. 5b) were significantly higher in the ZEEP group than in the sham group (*p* < 0.05), while infiltration was significantly higher in the ZEEP group than the PEEP group (*p* = 0.035). Hyaline membrane deposition (Fig. 5c) and edema severity (Fig. 5d) were both significantly higher in the ZEEP group compared to PEEP and sham groups (*p* < 0.05). Injury scores for all morphological features were positively correlated with average lactate-to-pyruvate ratio (Fig. 5e–h, Table 3). However, the correlation did not reach the level of significance for infiltration and alveolar damage scores.

### ICAM-1 and MPO Immunohistochemistry and Fluorescence microscopy

**Figure 6:**
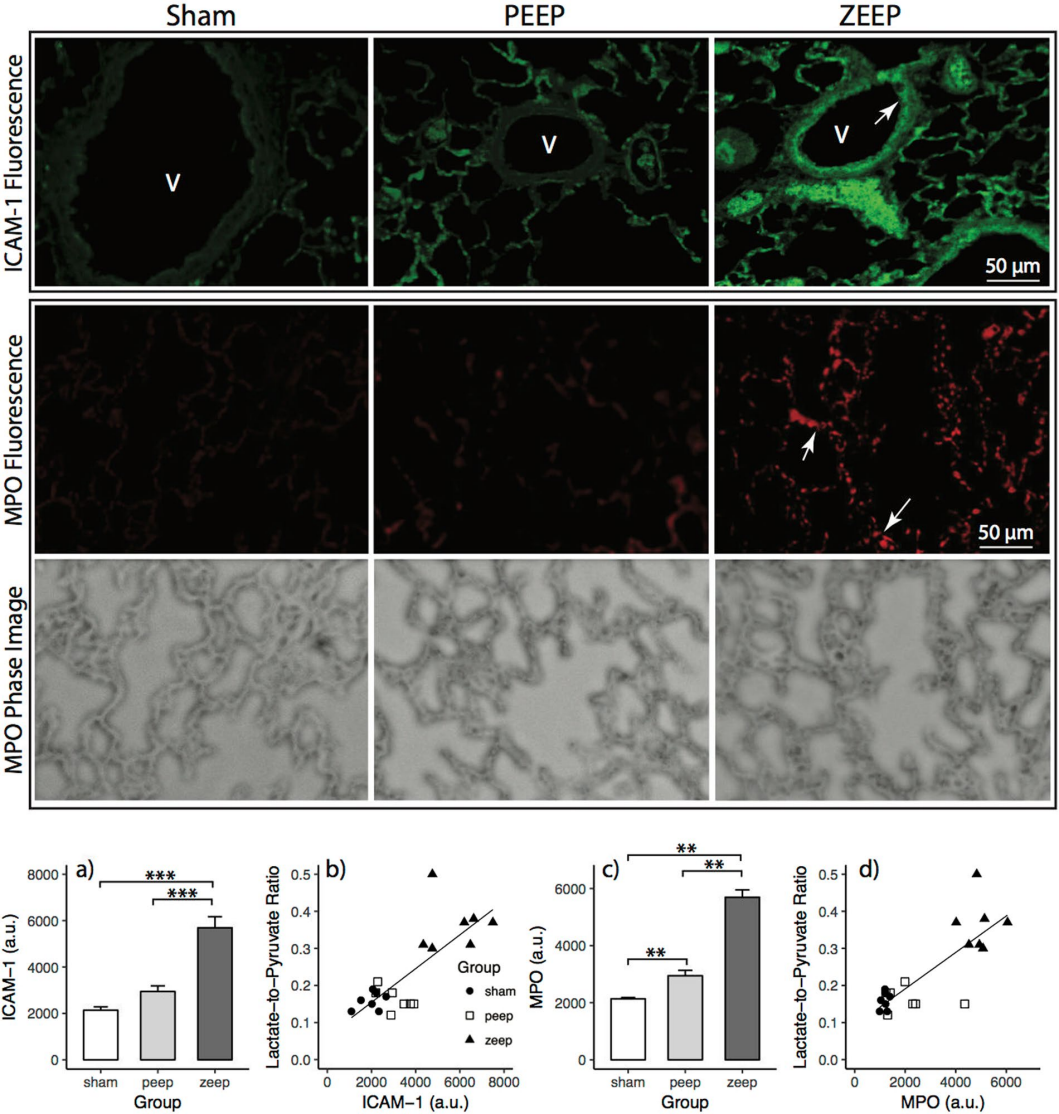
(Top) ICAM-1 expression in lung sections measured by fluorescence microscopy (40× magnification). Green fluorescence denotes ICAM-1 expression, which is higher in the ZEEP group in response to inflammatory stimuli. Additionally, the green fluorescence is higher along the endothelial layer that lines the vessels (indicated by white arrows (V)). (Middle) MPO expression in the lung sections measured by fluorescence microscopy (40× magnification). Red fluorescence denotes higher MPO expression, which is similar in both sham and PEEP lungs, but much higher in the ZEEP lungs. The white arrows illustrate the punctate staining from neutrophils. The phase images for the same sections show the structure of the lung tissue. The scale bar is 50 µm. (Bottom) ICAM-1 expression was significantly different among groups (F_2,18_ = 37.76, *p* < 0.001), and was significantly higher in the ZEEP group relative to the PEEP (p = 0.002) and sham (*p* = 0.002) groups (**a**). ICAM-1 expression was strongly correlated with the average lactate-to-pyruvate ratio measured 4 hours after acid/saline instillation (*r* = 0.782, *p* < 0.001) (**b**). There was significantly different MPO expression among groups (χ^2^(2) = 16.416, *p* < 0.001); the measured activity was significantly higher in the ZEEP group compared to the PEEP (*p* = 0.002) and sham (*p* = 0.002) groups (**c**), and was strongly correlated with the average lactate-to-pyruvate (*r* = 0.817, *p* < 0.001) (**d**). The activity was also significantly different between the PEEP and sham groups (*p* = 0.004). (**p < 0.01, ***p < 0.001).

ICAM-1 expression (Fig. 6a) was significantly different among groups (F_2,18_ = 37.76, *p* < 0.001), and was significantly higher in the ZEEP group compared to PEEP and sham groups (*p* = 0.002). ICAM-1 expression was strongly correlated with the average lactate-to-pyruvate ratio measured 4 hours after acid/saline instillation (Fig. 6b, Table 3).

There was significantly different MPO expression (Fig. 6c) among groups (χ^2^(2) = 16.416, p < 0.001): the measured activity was significantly higher in the ZEEP group compared to the PEEP and sham groups (*p* = 0.002), and was strongly correlated with average lactate-to-pyruvate (Fig. 6d, Table 3). The activity was also significantly different between PEEP and sham groups (*p* = 0.004).

### Regional analysis, lactate-to-pyruvate ratio

**Figure 7:**
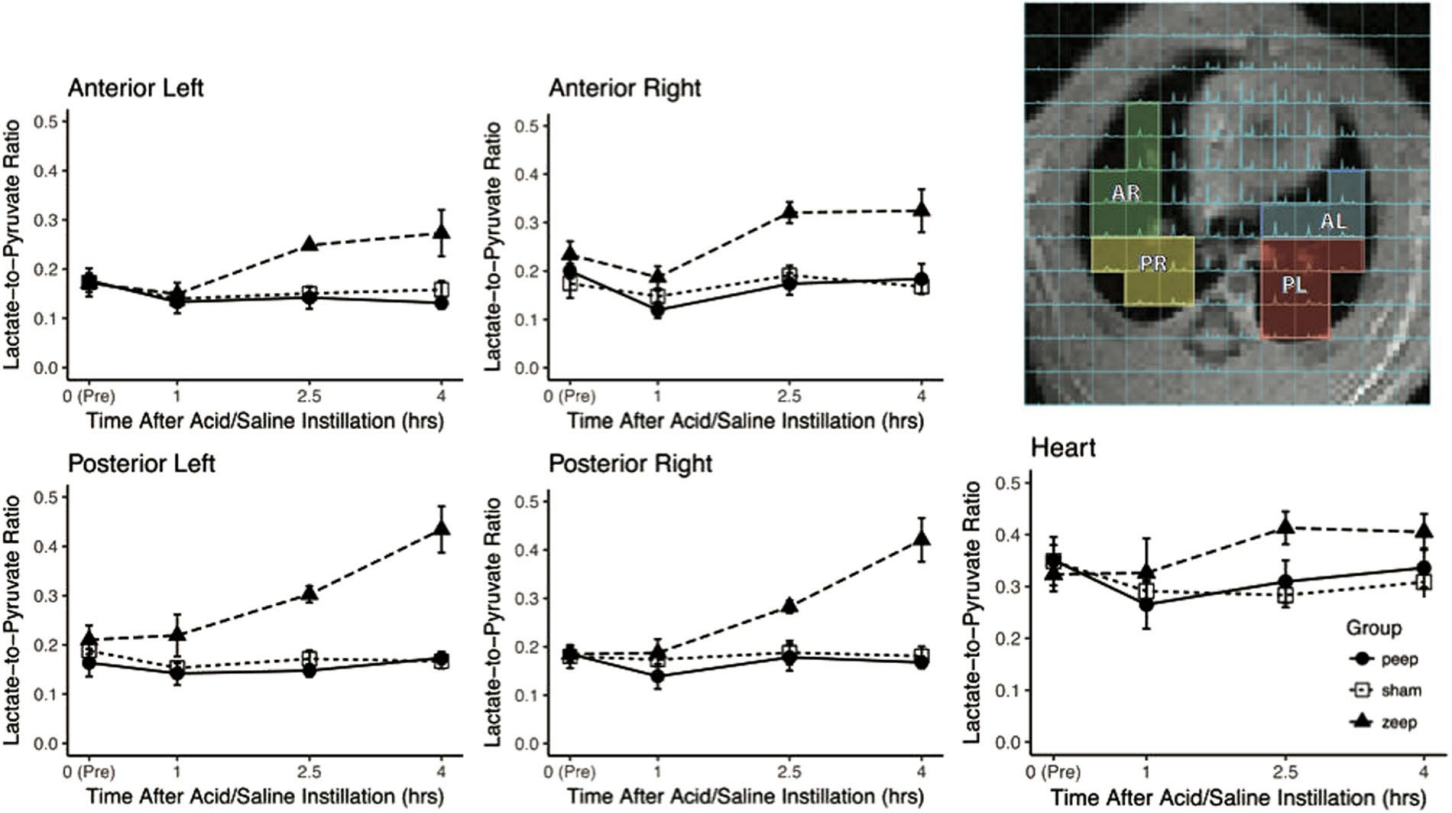
The right panel shows a selection of the voxels in the hyperpolarized carbon-13 spectroscopic image overlay. Lactate-to-pyruvate ratio was similar at baseline in all regions across all groups, and increases 2.5 hours after acid instillation in all lung regions in ZEEP rats. 4 hours after acid instillation, the ratio continues to increase in the posterior region on both sides. The lactate-to-pyruvate ratio measured in the myocardium and heart chambers remains unchanged over time in all groups (AL: anterior left, PL: posterior left, AR: anterior right and PR: posterior right).

At baseline, lactate-to-pyruvate ratio was similar in all regions (Fig. 7a–d). Moreover, it followed the same trend as the global lactate-to-pyruvate ratio shown in Fig. 3a: while it remained unchanged in the sham and PEEP groups, it increased 2.5 hours after acid instillation in the ZEEP group. The ratio further increased 4 hours after acid instillation in the posterior regions (Fig. 7b,d), but remained unchanged in the anterior regions (Fig. 7a,c). There was no apparent difference between the left and right sides. The lactate-to-pyruvate ratio measured over myocardium and cardiac chambers was similar among the groups, and remained unchanged over time (Fig. 7e).

## Discussion

In this study, we report the first *in-vivo* evidence that PEEP contains regional pulmonary anaerobic metabolism in an experimental model of lung injury (Figs 2 and 3a and Table 2). In contrast, we observed a progressive metabolic surge in the lungs when injured rats were ventilated without PEEP, permitting atelectasis. *Ex-vivo* histological and immunopathology assessment also supported a link between anaerobic metabolism and inflammatory activation in the lungs.

The investigation of altered pulmonary metabolism in the case of injury follows upon several ^18^F-FDG-PET showing increased glucose uptake in human and experimental ARDS^11,24,25^. Lactate is a major product of glucose utilization in the lungs^26^, and its increased production is an indication of the severity of lung injury in ARDS patients^15^. HP [1-^13^C] pyruvate MRI can measure regional lactate-to-pyruvate ratio, which reflects the endogenous pool of non-polarized lactate in the lung tissue^27^, thereby highlighting regional anaerobic glycolysis. Consequently, HP [1-^13^C] pyruvate MRI provides insight into the lung bioenergetics that is complementary to ^18^F-FDG PET, and can obtain such metabolic information within minutes without exposing patients to hazardous ionizing radiation. Perhaps most importantly, since hyperpolarization lasts for a significantly shorter time than the half-life of ^18^F-FDG, HP [1-^13^C] pyruvate imaging of the lungs may be repeated as frequently as desired to assess injury progression or treatment response. The latter is especially important in small animal research, where lung injury progression occurs on a time scale that is significantly shorter than in human patients.

In this study, we suggest a causal relationship between inflammation and increased HP lactate-to-pyruvate ratio, consistent with the link between increased glycolysis and the recruitment and activation of neutrophils as part of an innate inflammatory cascade proposed by previous FDG-PET studies^14^. However, other mechanisms may lead to increased lactate-to-pyruvate ratio as well: for example, stabilization of hypoxia-inducible factor 1-alpha (HIF1A) has been shown to occur during lung injury as a mechanism to attenuate inflammation, a process which also leads to elevated lactate dehydrogenase A (LDHA) expression in alveolar epithelial cells^28^. Nevertheless, it is unlikely that this was the dominant source of pulmonary lactate production in our study, as activated neutrophils are known to have significantly higher glycolytic rates compared to their neighboring cells^16,29^. Hypoxemia also leads to an increase in overall lactate levels in the blood and muscles^16,30^, and it is likely that regional tissue hypoxia due to injury progression can lead to increased pulmonary anaerobic metabolism. However, it is unlikely that hypoxia was the main cause of increased lactate-to-pyruvate ratio in our study: rats were not severely hypoxemic (S_p_O_2_ > 90%), and lungs are known to release normal levels of lactate under such conditions^31,32^. Furthermore, we did not observe any changes in cardiac metabolic activity (Fig. 7) suggesting absence of severe cardiac and systemic hypoxemia^33^. This may be due to the fact that the small dose of HCl used in this study caused relatively mild pulmonary injury^34^.

To determine the source of increased lactate-to-pyruvate ratio, we performed post-mortem histology and immunopathology measurements in lung tissue (Figs 5 and 6, Table 3). Overall, H&E staining showed formation of hyaline membranes and edema after injury (Fig. 5), which explains the trends of worsening lung mechanics (compliance) and lower oxygenation (Fig. 3b,c and Table 2). The decline in compliance and oxygen saturation in the ZEEP group during ventilation confirmed *in-vivo* progression of lung injury and atelectasis. Although compliance also declined over time in sham lungs, this was likely due to atelectasis caused by the absence of PEEP, as we previously documented in healthy lungs ventilated with similar settings^35^. However, this decline was not as significant in our study likely due the presence of intermittent sigh breaths in our protocol^36^. Additionally, the H&E staining showed widespread alveolar damage and influx of infiltrates into air spaces in both injured groups (Fig. 5a,b).

We further investigated the inflammatory status in these lungs by monitoring specific biomarkers of inflammation: the immunofluorescent staining patterns of the intercellular adhesion molecule-1 (ICAM-1) along the pulmonary vessel endothelial layer, and myeloperoxidases (MPO) in the entire lung section. While ICAM-1 is constitutively expressed in the lungs, its endothelial expression was significantly higher in the ZEEP group than in the PEEP and sham groups (Fig. 6), indicating enhanced endothelial activation that facilitates neutrophilic transmigration across the endothelium in the absence of PEEP^37,38^. Similarly, the significantly higher MPO expression in ZEEP lungs compared to both PEEP and sham lungs reflects enhanced endothelial activation^39^ and increased neutrophilic activity in the underlying tissue^38^. The dramatic concomitant increase in lactate-to-pyruvate ratio in the ZEEP lungs (Table 2), and its strong correlation with the ICAM-1 and MPO activity (Table 3), suggests that enhanced glycolysis and anaerobic metabolism in the absence of PEEP are associated with secondary inflammatory injury to the lungs.

Anaerobic metabolism was higher in the dependent (dorsal) regions of the ZEEP lungs, where proton density images showed more prominent radiological infiltrates (Fig. 7). Such gravitational distribution of the lactate signal suggests that atelectasis is a key factor in the genesis of abnormal metabolism at ZEEP. Studies using ^18^F-FDG-PET in ventilated animals reported high FDG uptake in dependent lung regions^40^, co-localized with atelectasis and inflammatory activation. In contrast, other small animal^10^ and human studies^11^ found alveolar injury^10^ and accelerated glucose metabolism^11^ in non-dependent lung regions exposed to high inspiratory stretch, rather than in atelectatic tissue. The latter findings are not necessarily in discordance with our results, however. In our previous work using hyperpolarized gas MRI in atelectasis-prone rat models, we detected elevated inspiratory stretch of residual ventilated airspaces in lung tissue with reduced gas content^41,42^, as well as higher stretch in dependent lung regions with more atelectasis^42^. Although we did not perform hyperpolarized gas MRI in the current study, the combined results of this and our previous work suggest that anaerobic metabolism and inflammation in the absence of PEEP may have been the result of augmented airspace stretch in lung regions with dependent atelectasis.

There are a number of limitations to our study. First, although the acid instillation model recapitulates ARDS, it is characterized by an initial epithelial injury and increased capillary permeability that is dependent on the instilled acid volume; this is followed by a secondary inflammatory response, but the extent of direct, immediate inflammatory action is minimized^34^. In our model, we limited direct injury by instilling only 0.5 ml/kg HCl, thereby allowing us to study tissue with a preponderance of secondary inflammation as it would occur during ventilation with ARDS. Second, we chose to monitor dynamic compliance instead of the static compliance in our study as a measure of injury progression. The former may be subject to bias due to alterations in airway resistance and intrinsic PEEP (a result of gas-trapping in the alveoli). However, in our previous studies we found this bias to be negligible^41,42^. The third limitation is that quantification of the absolute concentration of metabolites using hyperpolarized MRI is non-trivial, as the absolute signal level is subject to variability due to polarization level and physiological conditions^23^. Although the use of hyperpolarized lactate-to-pyruvate ratio as a surrogate for endogenous lactate concentration^15^ and glycolysis^18^ can mitigate this variability, it can still be subject to bias caused by excess fluid in the extracellular space resulting from capillary bed leakage^25^; the presence of such excess fluid can increase pyruvate concentration or limit its uptake, thereby reducing the lactate-to-pyruvate ratio and the sensitivity of the method^23^. PET imaging studies have addressed similar challenges by using compartment models to pinpoint the local source of signal^43^. In the case of MRI, this bias may be corrected for by using rapid MRI pulse sequences that allow the acquisition of multiple images to characterize metabolic flux in various tissues^18^. Finally, we only compared the averaged ICAM1 and MPO expression across the lungs with average lactate-to-pyruvate ratio, as co-localization of the images with histological slides was prohibitively difficult. Although the expression of these inflammatory markers is strongly correlated with global lactate-to-pyruvate, an even stronger local relationship between anaerobic metabolism and inflammation may be masked by a preponderance of non-injured lung regions.

In conclusion, we demonstrated the utility of hyperpolarized [1-^13^C] pyruvate MRI for assessing altered pulmonary anaerobic metabolism in an experimental model of aspiration pneumonitis, and showed that that PEEP contains anaerobic metabolism and secondary inflammation during the progression of lung injury. In contrast, we observed a substantial increase in anaerobic metabolic activity in the absence of PEEP, which was associated with neutrophil recruitment and accumulation. Although preliminary, these findings suggest a new approach to understanding the relationship between lung metabolism, lung damage, and inflammatory cascade during mechanical ventilation, and other conditions related to pulmonary disorders.

## Methods

### Animal preparation

Twenty-one male Sprague Dawley rats (306 ± 10 g, 8–10 weeks old) were used for this study (7 sham, 14 injured). All animal procedures were approved by the Institutional Animal Care and Use Committee (IUCAC) of the University of Pennsylvania (Philadelphia, PA) and were performed in accordance with relevant guidelines and regulations. All rats were diet-restricted to water only for 12 hours before the procedure to reduce inter-subject metabolic variability^44^. General anesthesia was induced by intra-peritoneal administration of sodium pentobarbital (40–60 mg/kg), the trachea was intubated with a 2-inch long, 14 gauge angiocatheter (BD, Franklin Lakes, NJ), and the glottis sealed (UHU Tac adhesive putty; Saunders Mfg. Co. Readfield, ME). Anesthesia was maintained with 1–2% isoflurane throughout imaging. All animals received IV hydration (3 ml.kg^−1^.hr^−1^). Body temperature was monitored using a rectal thermometer and maintained using warm air at 37 °C. Blood oxygen saturation and heart rate were monitored using an MR-compatible pulse oximeter (mouseOx, Starr Life Sciences, Oakmont PA). Animals were euthanized at the end of the experiment.

### Mechanical Ventilation and Lung Injury

Rats were ventilated using a small animal ventilator (VentElite, Harvard Apparatus, Holliston, MA) in the supine position (FiO_2_ 1.0, V_T_ 8 ml/kg, PEEP 5 cmH_2_O, Frequency 52 min^−1^). The ventilator embedded a sigh breath of 120% the tidal volume every ten breaths to prevent atelectasis. Airway pressure was monitored using an MR-compatible fiber-optic pressure sensor (FPI-HS, FISO, Quebec, QC). Primary lung injury was induced in 14 animals via intratracheal instillation of 0.5 ml/kg hydrochloric acid (HCl, pH 1.25) after healthy baseline imaging. HCl was injected in two equal aliquots, with the animal in the right and left lateral positions, respectively, and 45° head elevation. 7 sham rats received 0.5 ml/kg saline in a similar manner. Mechanical ventilation was resumed immediately after acid/saline instillation; for all animals, PEEP was set at 10 cmH_2_O for 10 minutes to allow the animals to stabilize, and then returned to 5 cmH_2_O. 7 animals with lung injury (PEEP group) remained on this ventilator setting until the end of the experiment (PEEP group). In the remaining seven injured animals (ZEEP group) and in the sham group, PEEP was reduced to 0 cmH_2_O 1 hour after acid/saline instillation. Peak inspiratory pressure (PIP) and dynamic compliance (C_dyn_ = V_T_/(PIP-PEEP)) of the respiratory system were obtained at ZEEP immediately before each image acquisition.

### Hyperpolarization

Pyruvate samples were prepared by mixing 14 M [1-^13^C] pyruvate (Cambridge Isotopes), 15 mM OX063 radical (GE Healthcare) and 1.5 mM of Dotarem (Guerbet LLC). 22 µL aliquots were polarized using a commercial HyperSense DNP polarizer (Oxford Instruments) for approximately one hour at ~1.4 K temperature. Samples were melted with 4 mL dissolution buffer (80 mM NaOH, 40 mM Trizma buffer, 50 mM NaCl and 0.1 mg/L EDTA) at 10-bar pressure and 180 °C to yield 80 mM isotonic neutral polarized [1-^13^C] pyruvate at 37 °C. Solid-state polarization was estimated to be 28 ± 3% for the studies based on the solid-state build-up curve and separate measurements. 4 ml/kg of the sample was administered through the tail vein over 6 seconds, followed by a 300-µL saline dose over 2 seconds to flush the pyruvate from the catheter’s dead volume. To minimize respiratory motion during data acquisition, a 12-second breath-hold was applied using the ventilator and data acquisition was initiated 18 seconds after the start of injection.

### MRI/MR Spectroscopic Imaging (MRSI)

Animals were imaged using a dual-tuned ^1^H/^13^C quadrature transmit/receive birdcage coil (m2m) in a 4.7 T horizontal-bore magnet (Varian Inc.). Axial and coronal proton images were acquired as anatomical references and to monitor the progression of injury using a multi-slice gradient echo sequence (TR/TE = 80/1.5 ms, α = 20°, 128 × 128 matrix size, 60 × 60 mm^2^ in-plane field-of-view (FOV), 100 kHz bandwidth and 16 averages). A total of sixteen 2 mm-thick slices were acquired to cover the lungs. Manual shimming was carried out at end expiration using a slice-selective pulse-and-acquire sequence over a 15-mm axial slice covering the lungs. Hyperpolarized [1-^13^C] pyruvate images were acquired using a custom-designed phase-encoded free-induction decay chemical shift imaging (CSI) pulse sequence to acquire 2D slice selective spectroscopic images^23^, (Imaging parameters: TR/TE = 35/0.5 ms, α = 9°, 16 × 16 matrix size, 128 spectral points, 4 kHz spectral bandwidth, 45 × 45 mm^2^ in-plane FOV, 15 mm slice thickness, 9-seconds acquisition time). Power calibration and frequency adjustment of the carbon-13 channel was performed using a 5-mm NMR tube containing 15 M solution of labeled ^13^C urea and 5 mM gadolinium (Gd) (Omniscan, GE Healthcare) placed next to the rat.

### MRI Data processing

All data were processed using custom routines programmed in MATLAB 2015b (MathWorks, Inc.). A 30 Hz exponential line broadening was applied to the individual FIDs. The spatially resolved spectra were computed by applying a 3D Fourier transform to the broadened data. The periodic acquisition of the k-space center (k_x,y_ = 0), performed by the custom-designed pulse sequence, was used to observe metabolite time-dependences and compensate for the signal loss due to T_1_ decay and other factors through amplitude normalization, as described in ref.^23^. First-order and local zero-order phase corrections were applied to the real part of the spectra, baseline correction was performed using 4^th^-order polynomial fit to the baseline for each spectrum, and the pyruvate peak was fit to a Lorentzian function. The linewidth and chemical shift estimates were used to fit individual Lorentzian functions to the metabolite peaks (lactate, pyruvate hydrate, bicarbonate and alanine). Approximately 10 voxels covering the lungs were manually selected from the MRSI/proton overlays to measure the global pyruvate and lactate values for the lung. Regional metabolism was quantified by manually selecting approximately 3 voxels from four different quadrants of the lungs (posterior/anterior and left/right). Average pulmonary lactate-to-pyruvate ratios were obtained by dividing the average lactate and pyruvate signals obtained from the selected voxels. We omitted quantification of alanine and bicarbonate metabolites in the lungs, as their intensities are too low for accurate quantification. Smoothed 64 × 64 metabolite maps were generated by integrating the area under the peak of the corresponding Lorentzian fits, and followed by spatial spline interpolation. Lung tissue was manually segmented from the axial proton images to generate a mask. The mask was applied to the smoothed metabolite and metabolite ratio maps, which were subsequently converted to DICOM format and overlaid on their corresponding proton images using OsiriX 8.1 (Pixmeo SARL, Switzerland).

### H&E, ICAM-1 and MPO Immunohistochemistry

After euthanasia, lungs were fixed in 10% formalin at 20 cmH_2_O pressure for 24 hours. Lungs were excised, sliced in axial plane, immunostained with hematoxylin and eosin (H&E) and assessed for injury by a pathologist. Each axial plane representing the whole lung was computationally sectioned into 10 sections, and sections were examined at 20×. Injury was assessed by a combination of infiltration (scale of 0–2: 0 = no infiltrate, 1 = infiltrate in the perivascular compartment, 2 = infiltrate in alveolar compartment), alveolar structure disruption (scale of 0–3: 0 = regular, 1 = distorted, 2 = collapsed with torn capillary–alveolar membrane, 3 = collapsed with opacity), hyaline membrane (scale of 0–5: 0 = None, 1 = detected in 1 or 2 areas, 2 = present in 3 or 4 areas, 3 = present in 5 or 6 areas, 4 = present in 7 or 8 areas and 5 = present in 9 or 10 areas) and edema (scale of 0–3: 0 = regular alveolus, 1 = slight thickening and 2–3 = dilated vessels in alveolar walls and proteinaceous material in alveolus). Additional slices were obtained to assess the expression of intercellular adhesion molecule-1 (ICAM-1) and myeloperoxidase (MPO), as reported earlier^45^: briefly, the paraffinized sections were deparaffinized in xylene and, after sequential ethanol and PBS wash, were immunostained with ICAM-1 and MPO primary antibodies with reactivity to rat species. The secondary antibodies used to measure the expression of ICAM-1 and MPO were a goat anti-mouse Alexa 488 (green) and goat anti-rabbit Alexa 594 (red), respectively, to measure inflammation and neutrophilic binding and activity in the lung tissue^46,47^. Fluorescence imaging was done using a Nikon fluorescence microscope (Nikon Diaphot TMD, Melville, NY). Images were acquired at excitation of 488 nm (for ICAM-1) and 594 nm (for MPO). All images were acquired at the same exposure settings (100 ms). ICAM-1 expression was quantified by integrating the fluorescence along the endothelial layer^48^. MPO expression was quantified by integrating the intensity of fluorescence across the entire field. For each rat lung section, at least 3–4 fields were imaged, and the data was quantified over these fields. All quantifications were performed using Metamorph Software (Molecular Devices, Downingtown PA) and ImageJ software (NIH).

### Statistical Analysis

The statistical analysis was performed using “R” (R Foundation for Statistical Computing, Austria, available at: http://www.R-project.org). Normality tests were performed using the Shapiro-Wilk test. Average lactate-to-pyruvate ratio and pulmonary compliance data were transformed to follow normal distributions via logarithmic and quadratic transformations, respectively. Statistical significance among injections and cohorts was tested using a two-way analysis-of-variance (ANOVA). Subsequently, statistical significance among groups was tested using a one-way ANOVA at each injection time point. Discrimination among the ICAM-1 fluorescence was measured using a one-way ANOVA. Post-hoc analysis was performed using Tukey’s honest significant test. A Kruskal-Wallis test followed by a Wilcoxon signed-rank *post-hoc* test was used when ANOVA conditions were not met (for S_p_O_2_, MPO and H&E histology scores). *α* = 0.05 was considered statistically significant. Holm-Bonferroni correction was applied when necessary. All data were expressed as mean ± SEM.

## Electronic supplementary material

Supplementary Information
