# Supplementary material for: Lung Metabolism and Inflammation during Mechanical Ventilation; An Imaging Approach
Source: Sci Rep. 2018 Feb 23;8:3525. doi: 10.1038/s41598-018-21901-0 (PMC5824838; doi:10.1038/s41598-018-21901-0)
Supplement: Supplementary file 1 — Supplementary Information [file 41598_2018_21901_MOESM1_ESM.docx]

**Lung Metabolism and Inflammation during Mechanical Ventilation; An Imaging Approach**

Mehrdad Pourfathi, Maurizio Cereda, Shampa Chatterjee, Yi Xin, Stephen Kadlecek, Ian Duncan, Hooman Hamedani, Sarmad Siddiqui, Harrilla Profka, Jason Ehrich, Kai Ruppert, Rahim R. Rizi

**Online Data Supplement**

**
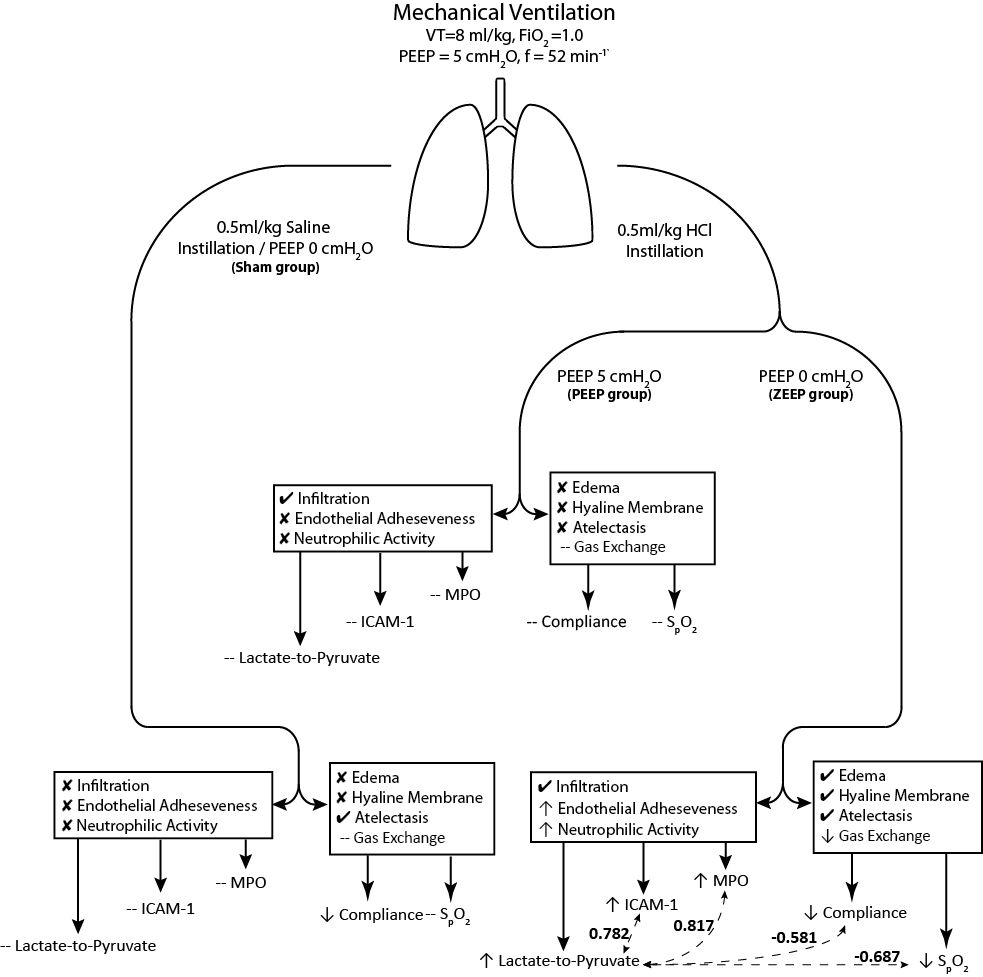
**

**Figure E1.** Acute lung injury was initialed via instillation of 0.5ml/kg HCl (pH 1.25) and was perpetuated in the ZEEP group by lowering the PEEP to 0 cmH_2_O; four hours after acid instillation (3 hours after PEEP was turned off), oxygen saturation and pulmonary compliance had declined significantly, and we measured elevated hyperpolarized lactate-to-pyruvate ratio. Post mortem study showed severe edema, hyaline membrane formation and atelectasis. Elevated ICAM-1 and MPO confirmed increased endothelial adhesiveness and the presence of activated neutrophils, respectively. Significant correlation between the lactate-to-pyruvate ratio, MPO and ICAM-1 suggests a causal relationship between increased anaerobic metabolism and inflammation. Injured lungs ventilated with PEEP (PEEP group) had no change in compliance and oxygen saturation. Despite the presence of neutrophil infiltration in the lungs, there was no change in pulmonary lactate-to-pyruvate ratio, ICAM-1 and MPO expressions, gas exchange or compliance—suggesting containment of inflammation. Finally, although compliance declined in rats instilled with saline—likely due to absence of PEEP—there was no change in the functional and cellular parameters observed in the study. ↑ = increase of; ↓ = decrease of; -- no change in; ✘absence; ✔presence; ICAM-1: intercellular adhesion molecule-1; MPO: myeloperoxidase, PEEP: positive-end expiratory pressure. (Numbers next to the arrows denote the correlation coefficient between lactate-to-pyruvate ratio and other parameters).

**
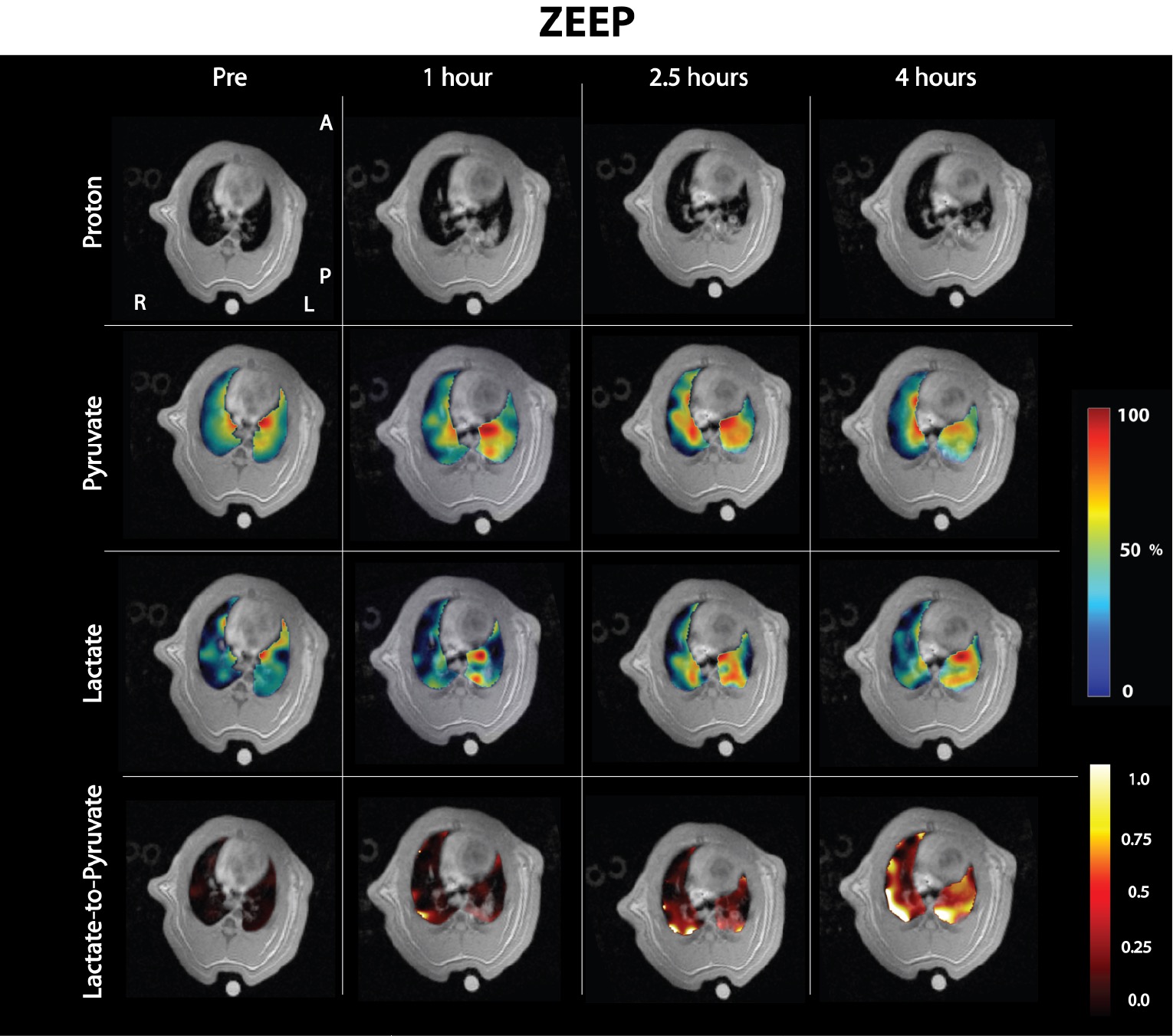
**

**Figure E2.** Representative proton images, pyruvate, lactate and lactate-to-pyruvate maps overlaid on their corresponding proton images for the ZEEP group at all four time points.

**
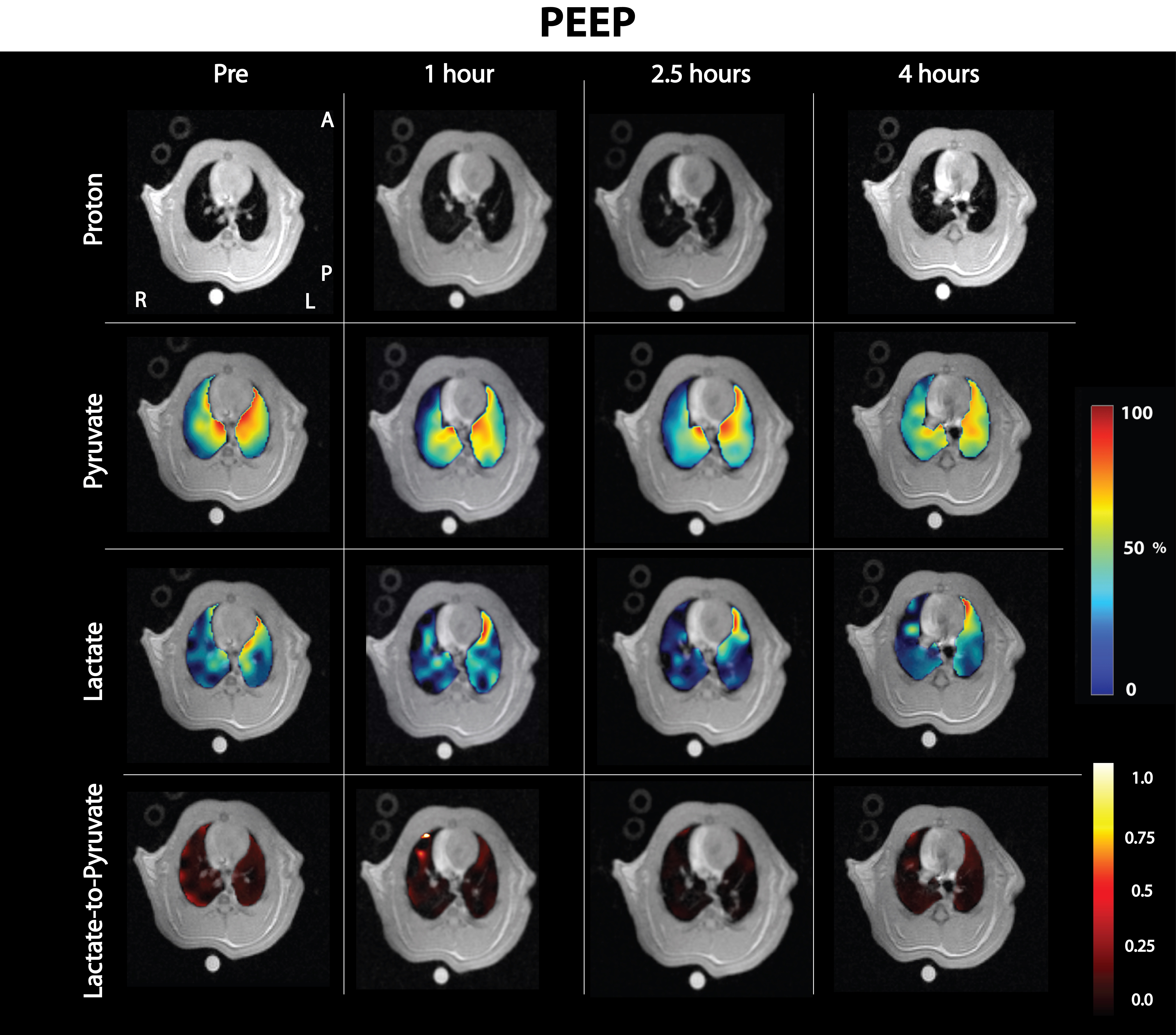
**

**Figure E3.** Representative proton images, pyruvate, lactate and lactate-to-pyruvate maps overlaid on their corresponding proton images for the PEEP group at all four time points.


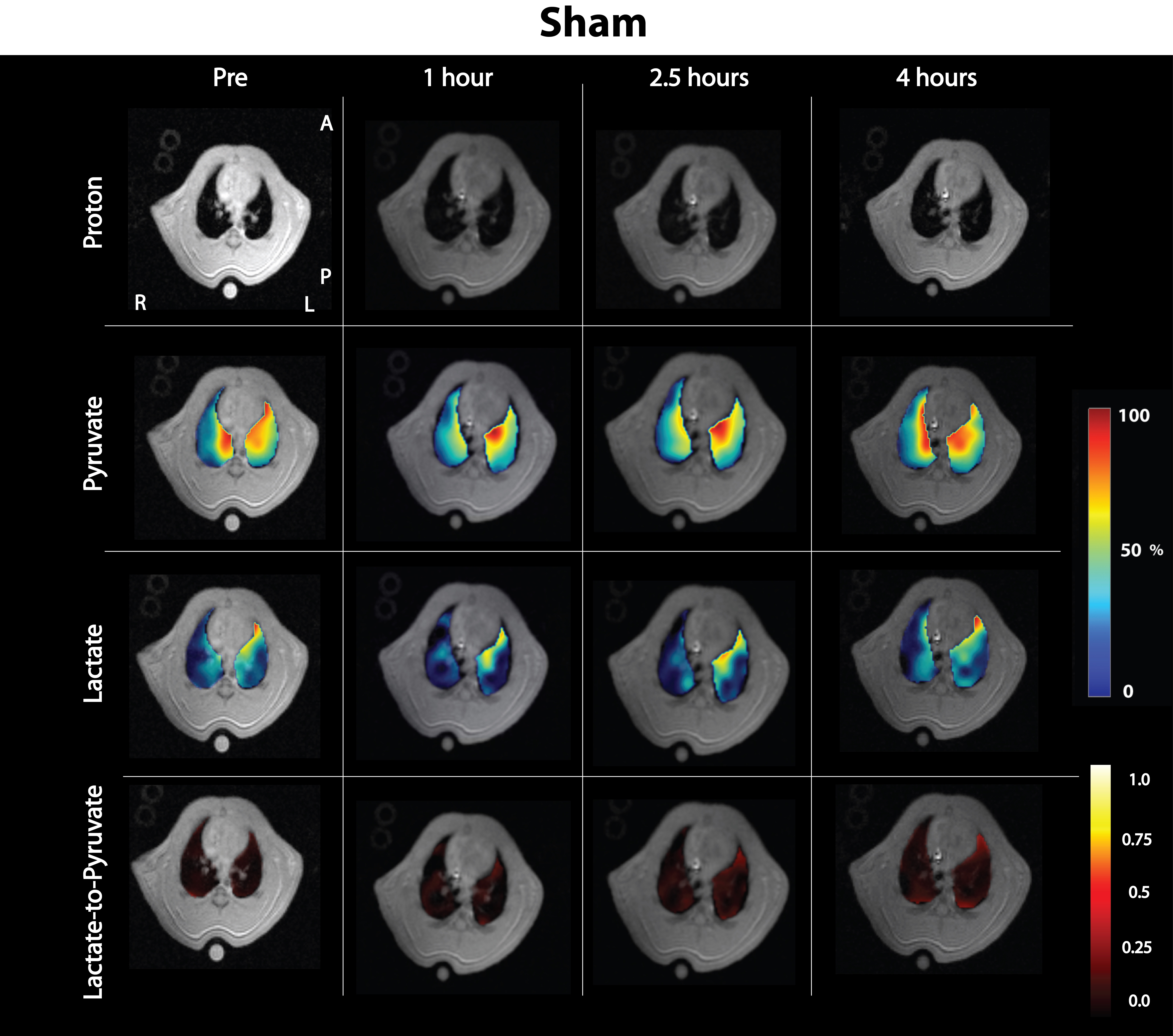


**Figure E4.** Representative proton images, pyruvate, lactate and lactate-to-pyruvate maps overlaid on their corresponding proton images for the Sham group at all four time points.

| **Cohort** | **Rat** | **Infiltration (0-2)** | **Alveolar Structure Damage (0-3)** | **Hyaline Membrane (0-5)** | **Edema (0-3)** | **Description** |
| --- | --- | --- | --- | --- | --- | --- |
| **ZEEP** | 1 | 1.5 | 1.8 | 5.0 | 2.6 | Inflammatory cell infiltration was observed in the alveolar cavity and pulmonary interstitium. In some regions, the alveolar structure is destroyed. Massive accumulation of erythrocytes observed in alveolar space. |
|  | 2 | 1.6 | 1.5 | 2.7 | 2.2 | Dilated vessels engorged with blood, collapsed and distorted alveoli, appearance of hyaline membrane. Infiltrates (seem to be a combination of macrophages and polymorphnuclear cells), alveolar septal thickening, and alveolar hemorrhage. The interstitial gap had widened in several regions. Appearance of membranous structures (possibly collagen fibers thus indicating onset of fibrosis) |
|  | 3 | 1.4 | 1.7 | 5.0 | 1.9 | Inflammatory cell infiltration, erythrocytes in alveolar spaces, congested capillaries, some destruction of alveolar structure |
|  | 4 | 1.5 | 1.7 | 5.0 | 1.6 | Extensive inflammatory cell infiltration, alveolar structure destroyed in large areas, appearance of membranous structures (possibly collagen fibers thus indicating onset of fibrosis), erythrocytes in alveolar space |
|  | 5 | 1.5 | 1.6 | 4.4 | 1.6 | Inflammatory cell infiltration, alveolar structure destroyed in large areas, appearance of membranous structures (possibly collagen fibers thus indicating onset of fibrosis), erythrocytes in alveolar space |
|  | 6 | 1.4 | 1.6 | 4.0 | 1.6 | Extensive inflammatory cells, blood in alveolus |
|  | 7 | 1.5 | 2.0 | 5.0 | 3.0 | Extensive inflammatory cell infiltration was observed both in alveolus and in the pulmonary interstitium. Collapse of the alveolar-capillary membrane. Proteinaceous debris in alveolus |
| **PEEP** | 1 | 1.3 | 1.0 | 2.7 | 0.8 | Some inflammatory cells, alveolar structure looks intact |
|  | 2 | 1.5 | 1.8 | 2.7 | 0.9 | Clumps of erythrocytes in capillaries, congested capillaries (possibly lung was not cleared of blood) |
|  | 3 | 1.6 | 1.6 | 3.3 | 0.8 | In general, alveolar structure is intact, some regions show inflammatory cells |
|  | 4 | 1.6 | 1.8 | 3.0 | 0.9 | Lots of erythrocytes in one region of the lung, the alveolar structure seems to be intact |
|  | 5 | 1.2 | 1.4 | 3.3 | 1.7 | Some inflammatory cells, alveolar structure looks intact |
|  | 6 | 1.3 | 1.6 | 3.0 | 1.3 | Some inflammatory cells; alveolar structure is collapsed in regions and minor edema is observed |
|  | 7 | 1.5 | 1.5 | 4.3 | 1.7 | Some regions show large number of inflammatory cells; alveolar structure is somewhat distorted but edema is not observed |
| **Sham** | 1 | 1.2 | 1.4 | 3.3 | 0.9 | Intact alveolar structure. Minor amount of blood in capillaries |
|  | 2 | 1.2 | 1.0 | 2.0 | 0.7 | Intact alveolar structure. Minor amount of blood in capillaries |
|  | 3 | 0.7 | 0.7 | 2.7 | 0.8 | Intact lung structure; no thickening or infiltration of cells |
|  | 4 | 1.0 | 1.1 | 2.3 | 1.0 | Normal structure, no thickening or anything |
|  | 5 | 1.3 | 1.6 | 2.7 | 0.6 | Normal structure |
|  | 6 | 1.0 | 0.7 | 2.0 | 0.6 | Normal lung, no infiltrate, no erythrocytes |
|  | 7 | 1.3 | 1.2 | 3.7 | 1.3 | Intact lung structure; no thickening or infiltration of cells |

**Table E1.** Summary of H&E Injury scores and pathological description
